# The value of fine needle aspiration cytology in the clinical management of rare salivary gland tumors

**DOI:** 10.1590/1678-7757-2017-0267

**Published:** 2018-02-15

**Authors:** Tibor Mezei, Simona Mocan, Alina Ormenisan, Beáta Baróti, Alina Iacob

**Affiliations:** 1 University of Medicine and Pharmacy of Tirgu Mures Department of Pathology Mures County Emergency Clinic Tirgu Mures Romania University of Medicine and Pharmacy of Tirgu Mures, Mures County Emergency Clinic, Department of Pathology, Tirgu Mures, Romania.; 2 University of Medicine and Pharmacy of Tirgu Mures Department of Oral and Maxillofacial Surgery Mures County Emergency Clinic Tirgu Mures Romania University of Medicine and Pharmacy of Tirgu Mures, Mures County Emergency Clinic, Department of Oral and Maxillofacial Surgery, Tirgu Mures, Romania.; 3 University of Medicine and Pharmacy of Tirgu Mures Department of Radiology Mures County Emergency Clinic Tirgu Mures Romania University of Medicine and Pharmacy of Tirgu Mures, Mures County Emergency Clinic, Department of Radiology, Tirgu Mures, Romania.

**Keywords:** Salivary gland neoplasms, Rare malignancies, Aspiration cytology, Cytopathology

## Abstract

Salivary gland tumors are relatively rare neoplasms, mostly located in the parotid gland, and few are malignant. Preoperative evaluation of salivary gland tumors includes fine needle aspiration cytology (FNAC).

**Objective:**

The purpose of this study was to determine the importance of FNAC in the evaluation of rare salivary gland neoplasms.

**Material and Methods:**

Four cases of rare salivary gland tumors were included, which were preoperatively assessed by clinical investigation, computed tomography, and FNAC.

**Results:**

The presented cases include myoepithelial carcinoma, oncocytic carcinoma, undifferentiated lymphoepithelial carcinoma, and marginal zone lymphoma.

**Conclusion:**

FNAC is a reliable diagnostic tool for common salivary gland neoplasms; however, rare tumors often represent diagnostic challenges.

**Clinical relevance:**

In such rare tumors, the role of aspiration cytology may be limited to establishing the dignity of the lesion (benign/malignant). This knowledge enables the surgeon to choose the most appropriate therapeutic procedure. A definitive diagnosis of rare tumors (either epithelial or nonepithelial) is obtained by histological examination; cytology is limited in this regard due to overlapping features.

## Introduction

Salivary gland tumors are relatively rare neoplasms, with an incidence of about 2.5-3 cases *per* 100,000, and represent about 3% of all head and neck tumors. Most salivary gland tumors are in the parotid gland (75-80%) and only 20% of them are malignant^5^^,^^26^. Etiology of salivary gland tumors is still under debate; it is presumed that smoking, viral infections, and genetic predisposition may all play a significant role in their pathogenesis. Ionizing radiation is probably the only well-established risk factor with documented role in the etiology of these tumors^15^^,^^32^.

The initial preoperative evaluation of salivary gland tumors includes patient history, followed by thorough clinical examination of the face and neck, imaging tests (especially ultrasonography and computed tomography), and fine-needle aspiration cytology (FNAC). FNAC is a preferred method for obtaining morphological diagnosis prior to surgery. It is relatively low cost, less invasive, and has the ability to offer preoperative diagnosis, which makes it a preferred method of diagnosis. FNAC is effective in providing rapid morphological assessment of lesions, offering valuable information about the origin (salivary or non-salivary), nature (benign or malignant) and/or grade (low or high grade) of the tumors. Information thus obtained is an invaluable aid in proper clinical and surgical management^4^^,^^6^.

Although this is valid for most cases, cytology alone, however, cannot provide a definitive diagnosis in some cases where histological diagnosis is the gold standard. FNAC has limitations, especially in identifying rare entities and distinguishing lesions that have overlapping cytological features, which is often the case in salivary gland tumors^3^.

The World Health Organization classifies salivary gland epithelial tumors into 10 benign and 23 malignant entities. Non-epithelial neoplasms are rare, representing about 2-5% of all salivary gland tumors. This complex classification is based on histological features and has the advantage of estimating the prognostic and therapeutic outcomes based on the different behavior of each tumor type. The great diversity of these tumors is related to various cell types found in salivary glands (ductal, acinar, myoepithelial, and basal cell types)^5^. This plethora of diverse types, however, also turns FNAC diagnosis into a challenging task.

The purpose of this study was to highlight the role of FNAC in the evaluation and clinical management of rare salivary gland neoplasms, with emphasis on a multidisciplinary approach.

## Material and methods

Four patients included in this study were hospitalized in the Department of Oral and Maxillofacial Surgery of the Mures County Emergency Clinic (Spitalul Clinic Judetean de Urgenta Mures); cytological and histological diagnosis were conducted at the Pathology Laboratory of the same hospital. Upon submission, a signed written informed consent was obtained from all four patients. No additional tissues/cells were removed from any patient, other than what was necessary for accurate cytological/histological diagnosis.

Cytological material was obtained using standard FNAC technique. An average of three passes *per* patient was performed, each resulting in one or two smears. A Cameco-type syringe holder fastened with a 10-ml three-piece syringe and a 23G needle was used. The aspired material was spread on standard pre-cleaned glass slides (Menzel-Glaser Superfrost, Braunschweig, Germany) using the standard one-step method. Wet-fixation (immediate immersion into 95% ethyl alcohol) and standard Papanicolaou staining was used. Histological specimens were prepared from surgically removed tissues. Standard hematoxylineosin staining was applied, with the use of additional immunohistochemical markers, as mentioned later.

The study complies with the recommendations of the Helsinki Declaration guidelines, and it was approved by the ethics committee of our university. The identity of the patients was protected at several levels. From their clinical data, only tumor localization, size, age, sex, cytology diagnosis, and histopathological type were used. Patient confidentiality was respected by anonymizing biopsy specimens.

## Results, case series

### Case 1

**Figure 1:**
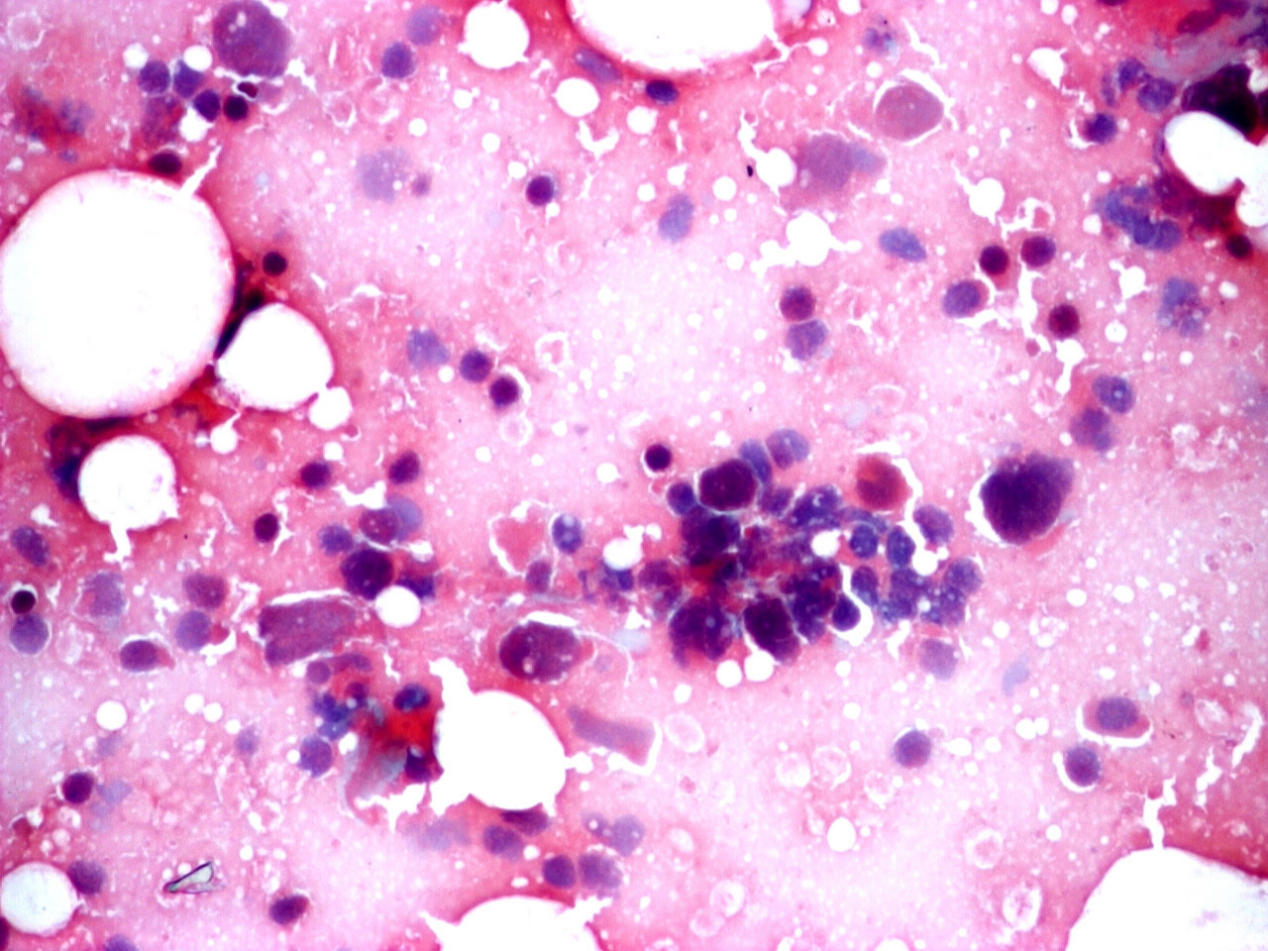
Myoepithelial carcinoma, cytology. Smears have rich cellularity and are dominated by polygonal pleomorphic tumor cells with abundant delicate cytoplasm. Nuclear atypia in the form of hyperchromasia, coarse chromatine distribution, and occasional large nuclei are observed. Neoplastic cells occasionally form cohesive clusters, and isolated cells are frequently seen. Cell non-neoplastic small mature lymphocytes are also observed. Background is hemorrhagic. (obj. 20×, Papanicolaou stain)

**Figure 2:**
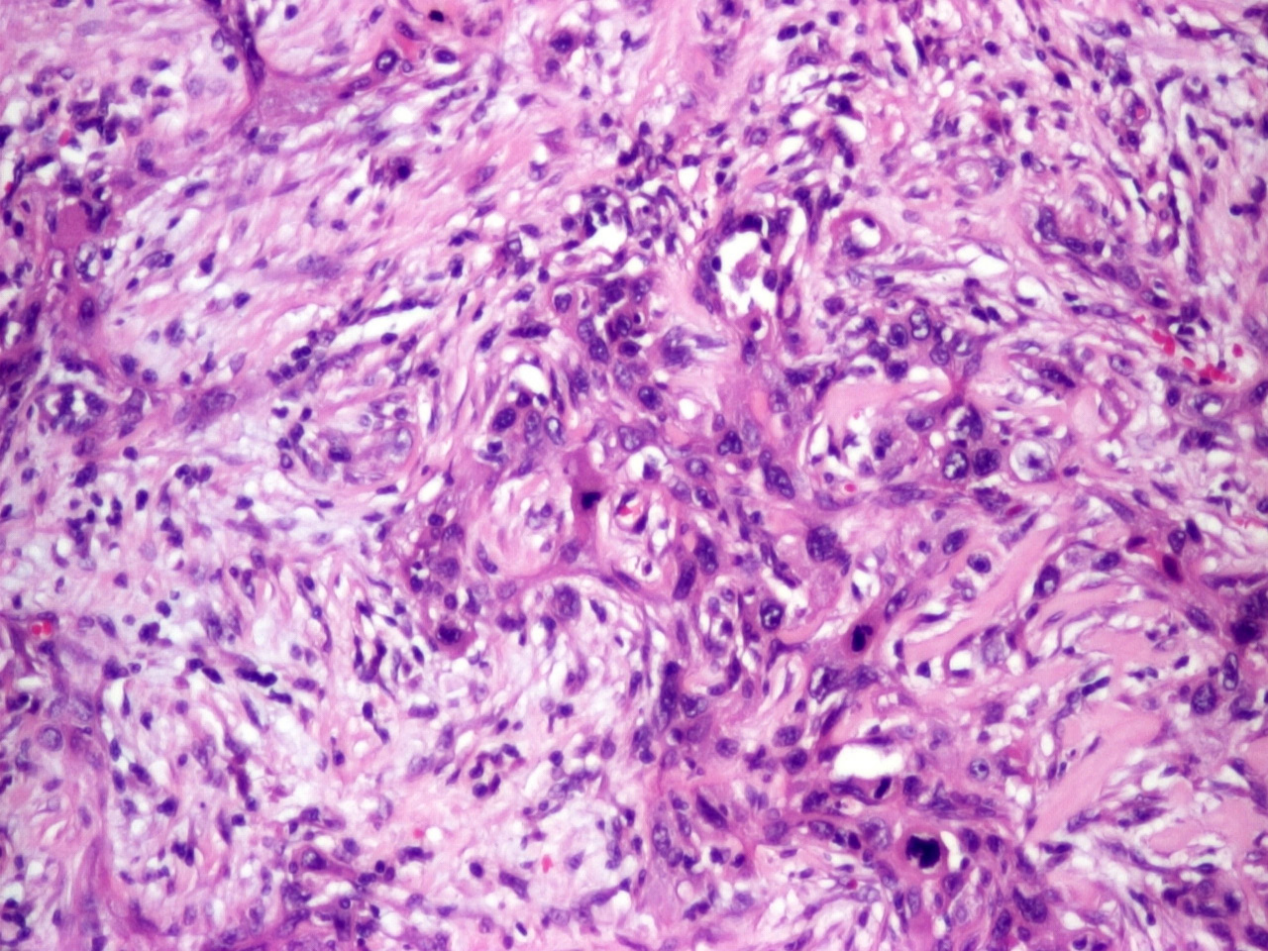
Myoepithelial carcinoma, histology. The tumor shows a somewhat nodular and solid growth pattern. Tumor cells exhibit clear cut pleomorphism; many of them showing epithelial morphology or fusiform shape, vacuolated cytoplasm with eosinophilic and some myxoid material in the background. (obj. 20×, hematoxylin and eosin stain)

A 92-year-old male patient presented at the Department of Oral and Maxillofacial Surgery due to a right parotid painless mass, clinically suggestive of a malignant neoplasm, present for the past six to seven months, with recently developed facial paresis. Physical examination showed a tumor of about 6 cm in its greatest diameter, with diffuse, irregular palpatory appearance, fixed to underlying structures, covered by normal skin. Computed Tomography (CT) showed a tumor mass extending into the right parotid gland parenchyma, without invasion of adjacent maxillary structures and lateral-cervical lymphadenopathy. Preoperative FNAC resulted in moderately cellular smears, composed of polymorphous lymphocytes and many atypical cells with large, hyperchromatic nuclei, some lacking cytoplasm, suggesting a malignant neoplasm. Atypical cells displayed characteristics of epithelial cells with moderate atypia (Figure 1). Relatively scant stromal elements were seen. No specific features of common entities were observed; however, the malignant nature of the lesion was obvious. The patient underwent total parotidectomy with facial nerve resection because intraoperatively the nerve was found to be embedded into the tumor. Histopathology showed a multinodular neoplastic process with infiltrative margins in the right parotid parenchyma, with the effacement of normal ductoacinar structures. A proliferation of fusiform cells, some displaying epithelioid features, with marked nuclear pleomorphism and atypical mitoses, were seen. Markedly hyaline stroma and widespread areas of the perineural invasion were also present (Figure 2). Tumor cells displayed positivity for cytokeratins 5/6 and 7, smooth muscle antigen (SMA), S100 and p63 proteins, and were negative for desmin, thus indicating the myoepithelial origin. A diagnosis of the myoepithelial carcinoma was made, and the patient was referred to the Oncology Department for further treatment; but unfortunately, he refused any further therapy, and has no longer presented for clinical follow-up.

### Case 2

**Figure 3:**
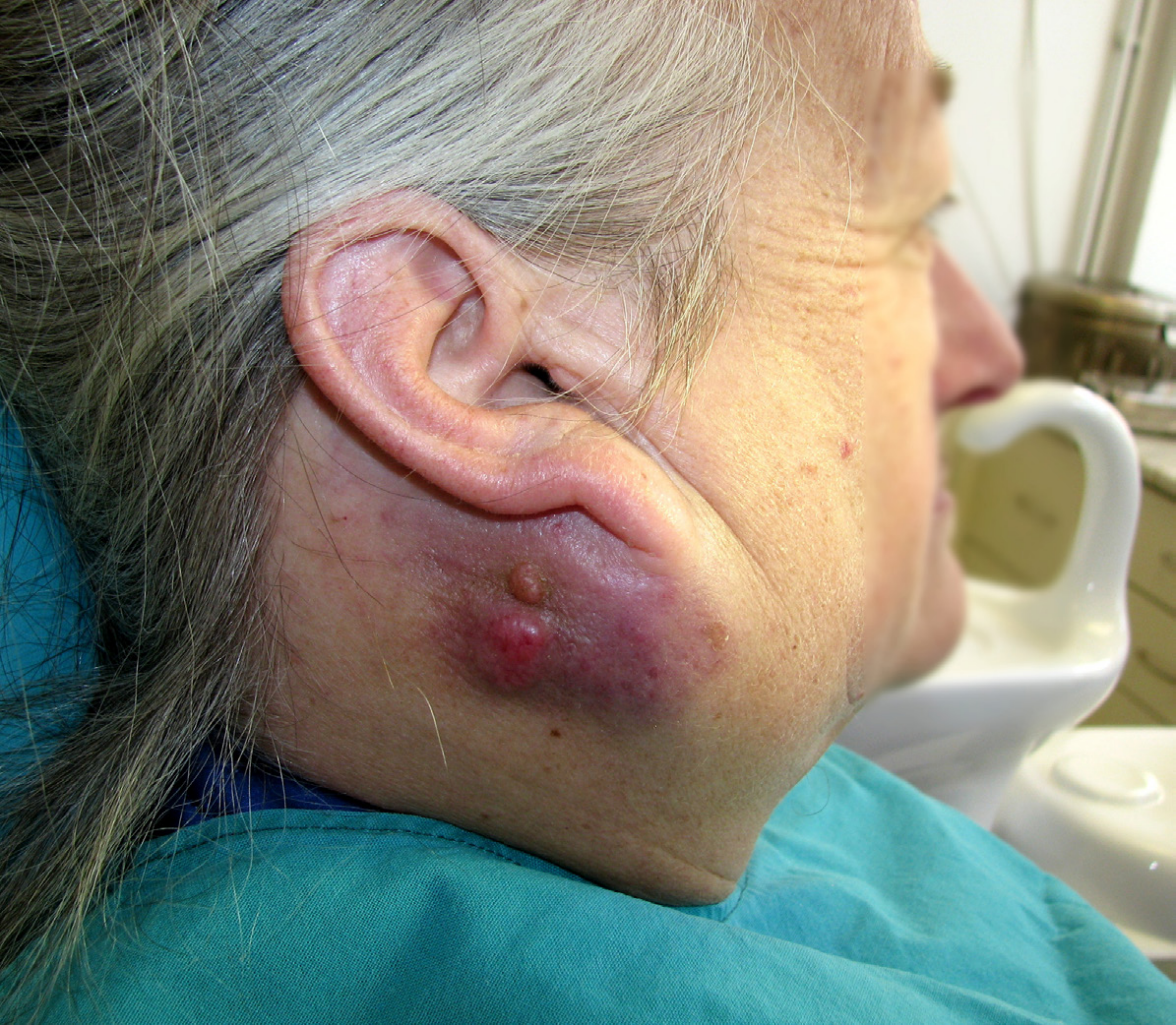
Oncocytic carcinoma, clinical aspect. A right-sided subcutaneous inferior retro-auricular mass is observed, with a somewhat bosselated surface. Overlying skin is shiny and shows a bluish red discoloration. (Face partially blurred for patient privacy)

**Figure 4:**
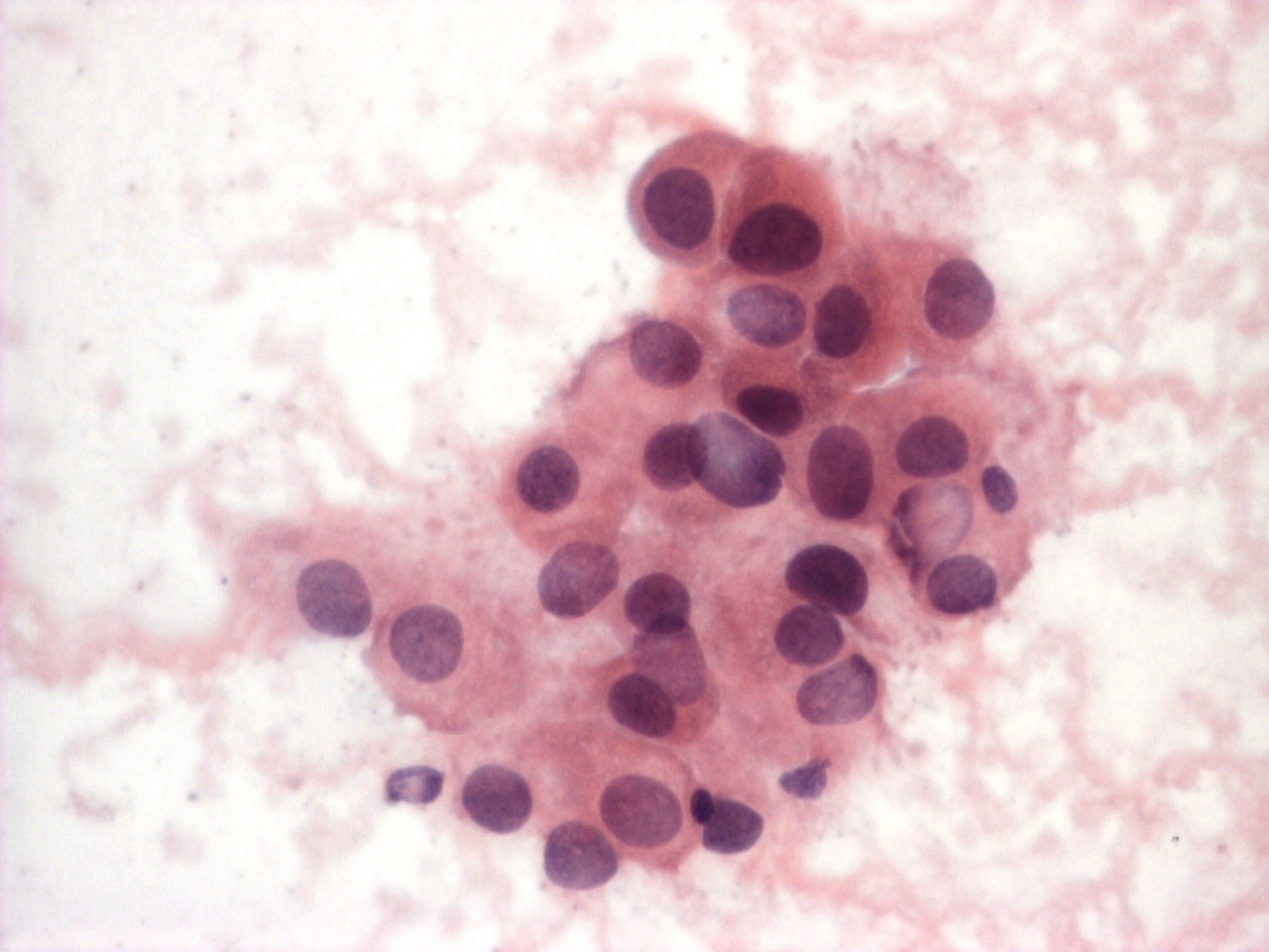
Oncocytic carcinoma, cytology. A group of oncocytic cell is observed. The cells show moderate nuclear atypia (hyperchromasia, slight nuclear membrane irregularities, anisonucleosis). The cells have abundant finely granular eosinophilic cytoplasm, nuclei are eccentrically located (obj. 40×, Papanicolaou stain)

A 62-year-old female was referred to the clinic for a painless, slow growing, upper right lateral-cervical nodular mass. Physical examination showed a firm, fixed mass, measuring about 10 cm in greatest diameter, located at the inferior pole of the right parotid gland without facial nerve involvement or lymph node enlargement (Figure 3). The patient had congestive heart failure, atrial fibrillation, and chronic obstructive pulmonary disease. Computed tomography described the tumor as extending to the upper third of the lateral-cervical region and inferior pole of the right parotid, with extension to the sternocleidomastoid muscle and the carotid-jugular vascular bundle and neighboring structures. FNAC showed numerous neoplastic cells with oncocytic change (finely granular eosinophilic cytoplasm, well defined cytoplasmic borders, slightly discohesive, moderately eccentric nuclei), many nuclei lacking cytoplasm, with moderate nuclear atypia and an associated but reduced lymphocytic population (Figure 4). It was diagnosed as an oncocytic tumor. The patient's severely altered general condition (stage IV tumor), associated diseases (chronic cardiac failure, chronic obstructive pulmonary disease) precluded a more aggressive surgical approach. In this setting, a FNAC followed by incisional biopsy was performed.

Histological examination revealed muscular, connective, and fatty tissues infiltration by proliferating tumor cells, tumor cells arranged in solid structures without secondary lumen formation, occasionally with discohesive growth pattern with foci of perineural invasion. The tumor cells were large, with abundant, finely vacuolated, eosinophilic cytoplasm, with large central, vesicular nuclei, and prominent, eosinophilic nucleoli. Numerous mitoses and a relatively abundant inflammatory infiltrate were observed. Tumor cells expressed cytokeratins AE1/AE3 and 7, and were negative for cytokeratin 20, S100 protein, and HMB45. The final histological diagnosis was oncocytic carcinoma. The patient refused surgery due to her associated diseases and was referred for palliative oncology. She underwent adjuvant radiotherapy – 70 Gy in 35 sessions. She presented only once for clinical follow-up, one month after radiotherapy. Her general condition had improved slightly.

### Case 3

**Figure 5:**
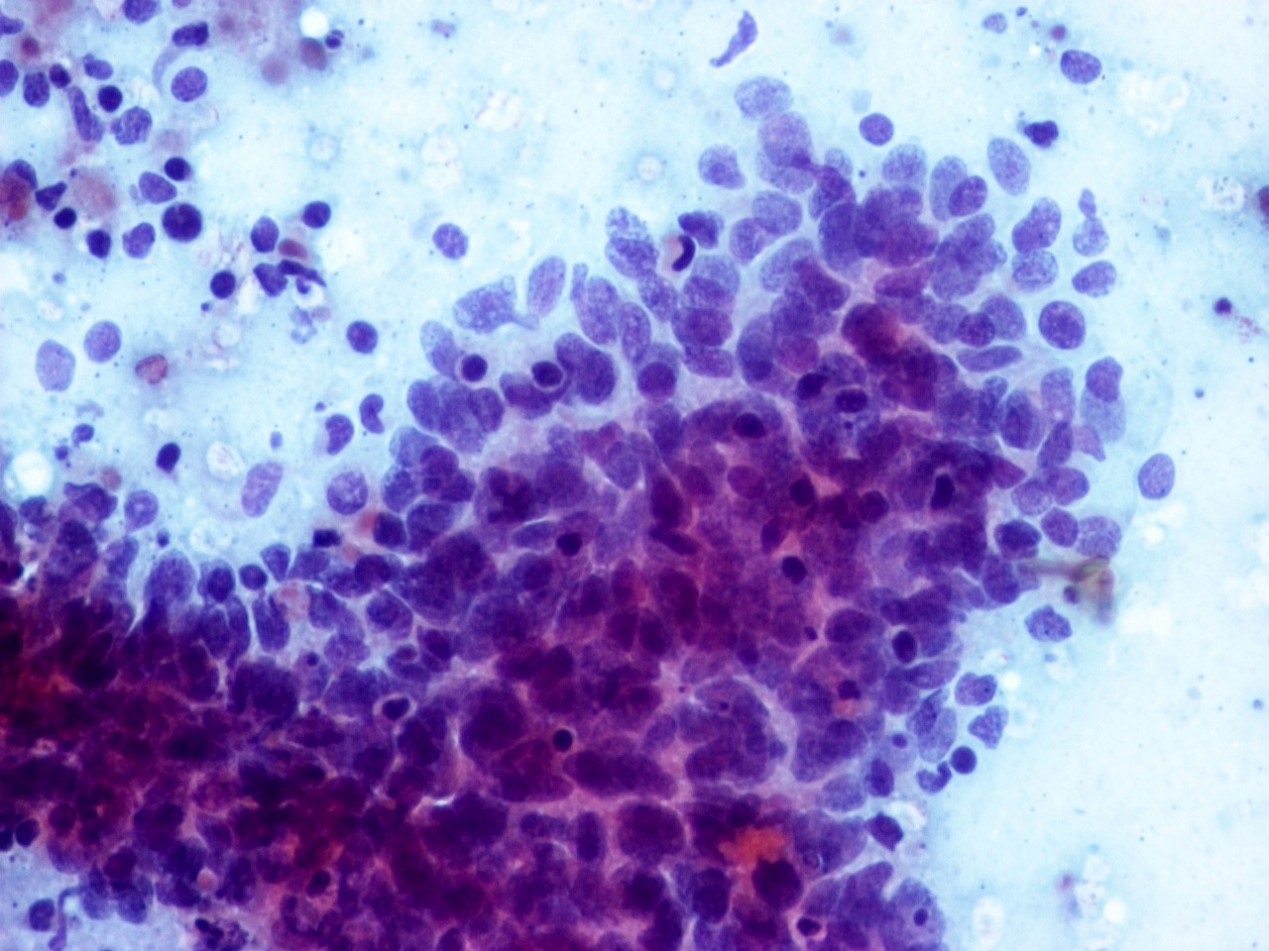
Undifferentiated lymphoepithelial carcinoma, cytology. Smears showed high cellularity composed of aggregates of atypical epithelial cells in a background of mature lymphocytes. Neoplastic cells are predominantly found as cohesive clusters with scattered isolated cells at the periphery. Atypical cells having poorly defined cytoplasmic borders, large vesicular nucleus, finely granular chromatin, occasionally with single prominent nucleolus. (obj. 20×, Papanicolaou stain)

A 66-year-old male patient presented with facial asymmetry caused by a painless mass on the right parotid area, discovered a year ago, that showed accelerated growth in the past four months. Clinical examination revealed a firm, irregular, 5 cm diameter mass arising in the right pre-auricular region, fixed to deeper tissues and overlying skin, without clinical evidence of facial nerve involvement. Ultrasonography showed a hypoechogenic mass of 40×33 mm with irregular contour, heterogeneous structure, and rich vascular image, confirmed by computed tomography as well, without lymph node involvement. FNAC described atypical epithelial cells with large, hyperchromic nuclei in a rich lymphocytic background and many histiocytes of foamy cytoplasm with tumor diathesis. A diagnosis of undifferentiated carcinoma was assessed on the cytological examination (Figure 5). Two surgical interventions were performed. Firstly, since no pathological lymph node enlargement was observed on initial CT scan, total parotidectomy was performed. Superior lateral-cervical lymph node enlargement was observed six months after the initial surgery, and was confirmed by subsequent CT scan. Right selective neck dissection was performed in accordance with current protocols for the treatment of malignant parotid tumors.

**Figure 6:**
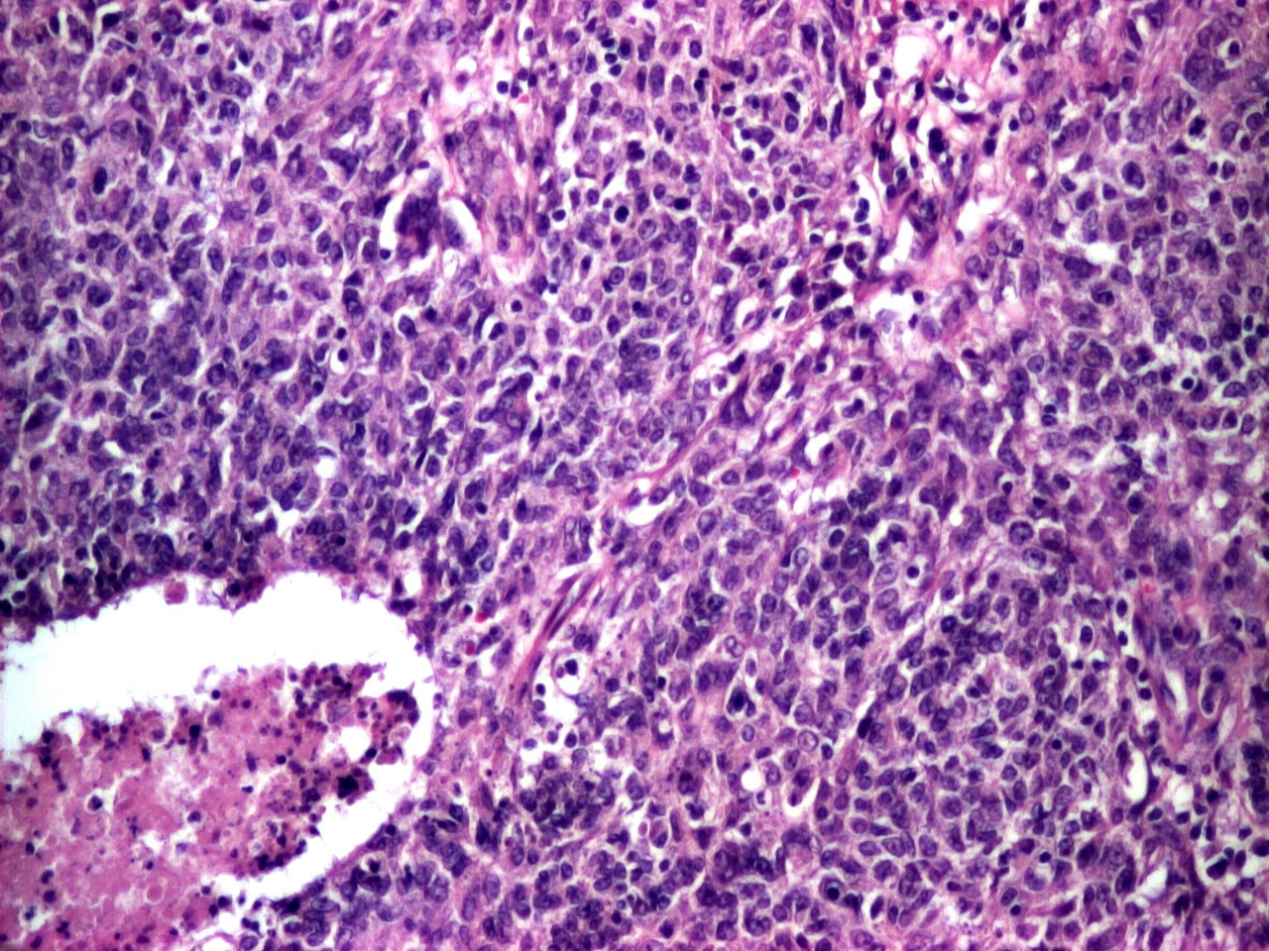
Undifferentiated lymphoepithelial carcinoma, histology. Proliferation of large undifferentiated epithelial cells are observed, forming large solid sheets. No glandular or squamous differentiation is observed. A fine vascular stroma is present with lympho-plasmocytic infiltration. Necrosis is also evident. (ob. 10×, hematoxylin and eosin)

Histopathology revealed a tumor consisting on large clusters of atypical cells with indistinct margins, frequently displaying syncytial appearance. The tumor cells had scanty cytoplasm, large, vesicular nuclei, and prominent nucleoli. Numerous atypical mitoses were seen. The cellular islands showed central necrosis and abundant inflammatory infiltrate at the periphery. Small mature lymphocytes were also found scattered among tumor cells (Figure 6). Residual parotid parenchyma containing two lymph nodes, one with tumor invasion, was also present. The tumor presented lymphovascular and perineural invasion. Tumor cells were positive only for cytokeratin AE1/AE3, and negative for cytokeratin 5/6 and 7, p63, SMA, PS100, chromogranin, and synaptophysin. The final diagnosis was undifferentiated lymphoepithelial carcinoma. The patient underwent radiotherapy (70 Gy in 35 sessions), returned for regular clinical checks every 3 months in the first year, and then disappeared. During these visits, his general condition seemed to have considerably improved.

### Case 4

**Figure 7:**
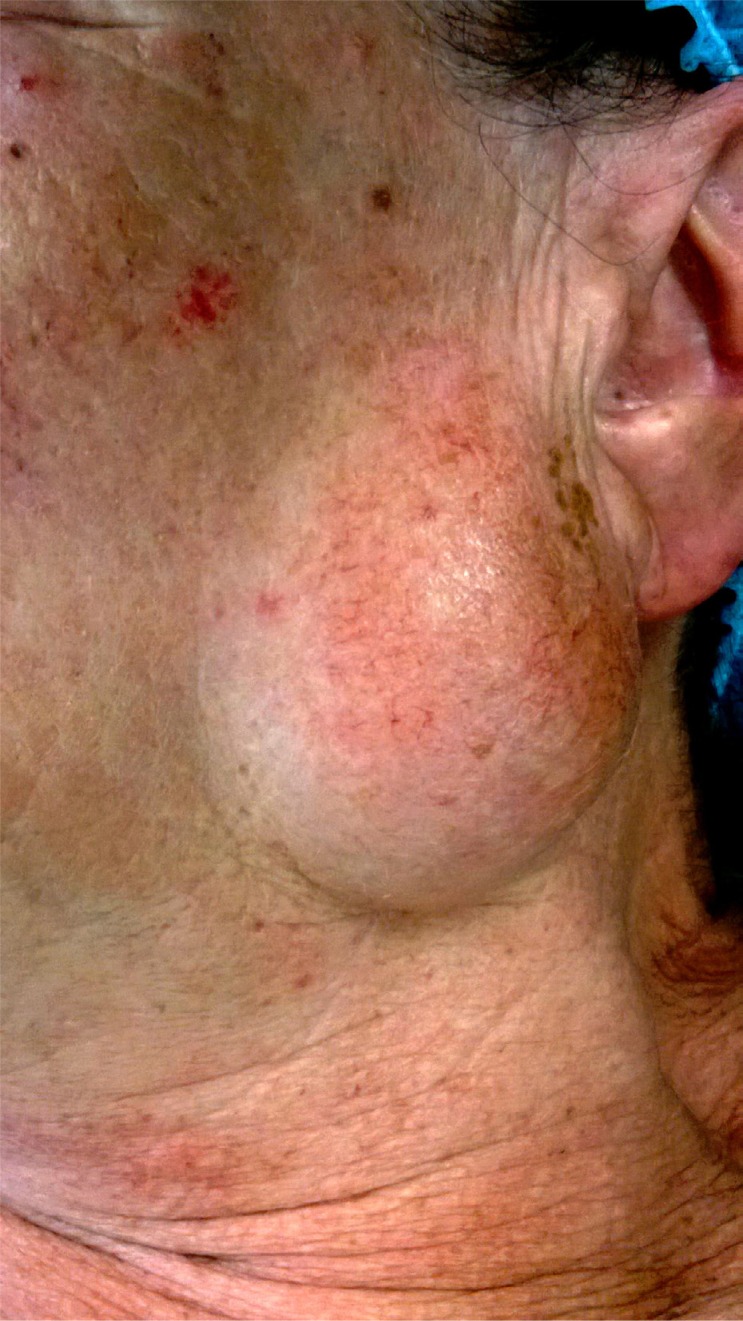
Marginal zone B-cell lymphoma, clinical aspect. A left-sided, relatively well-demarcated preauricular, firm, non-tender mass are observed. Overlying skin is shiny

**Figure 8:**
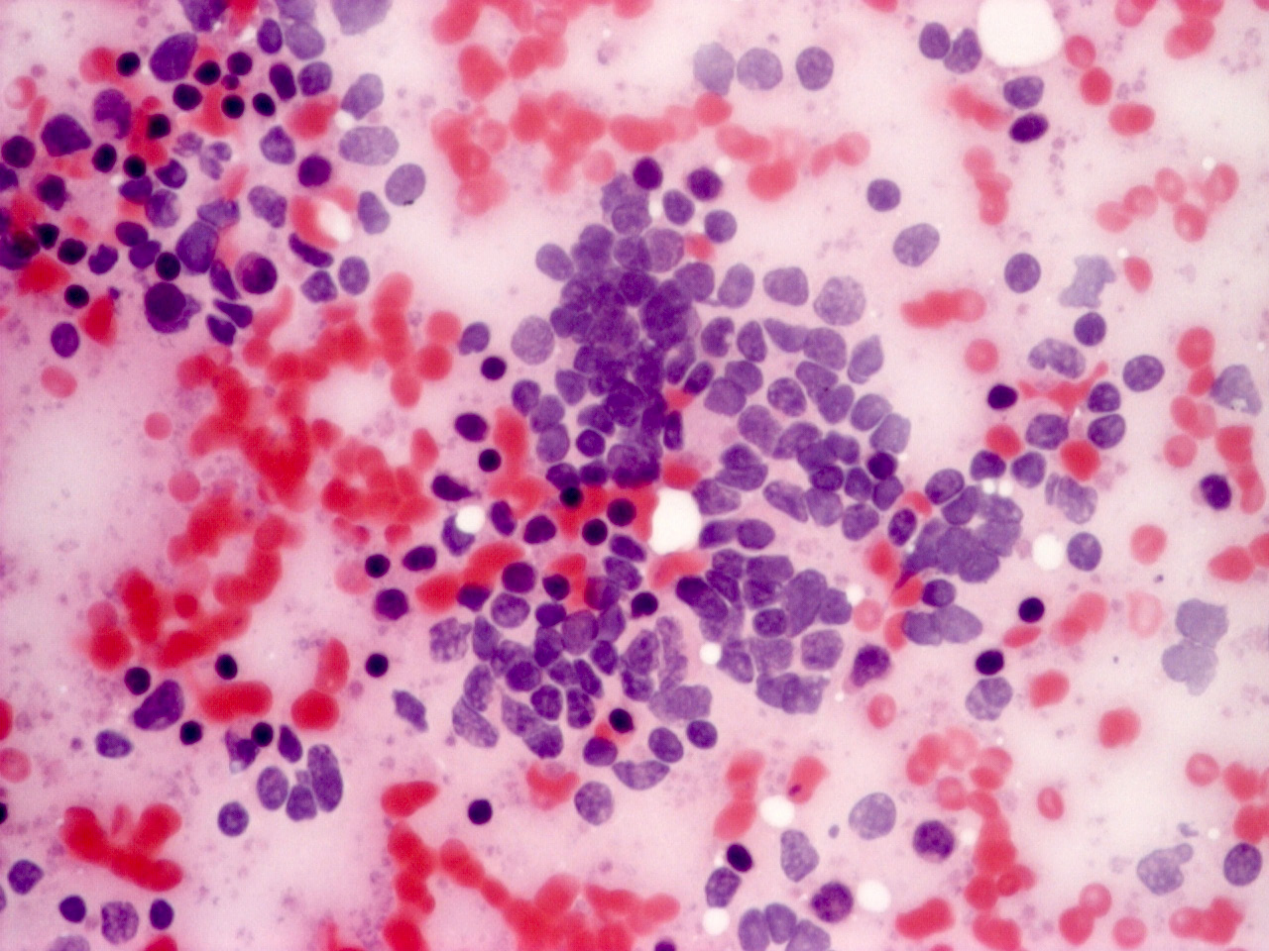
Marginal zone B-cell lymphoma, cytology. Smears are composed of two distinct lymphoid cell populations, both having indistinct cellular margins. One is composed of medium sized, containing relatively large nucleus, with atypical nuclear borders, occasional nuclear molding, irregular chromatin, occasional large nucleoli. The other consists of normal looking, small mature lymphocytes. Neoplastic cells apparently are clustered, however a propensity for decohesion is readily observed (obj. 20×, Papanicolaou stain)

A 73-year-old female patient was examined at the outpatient clinic with a right facial painless mass, showing accelerated growth in the past two months, that has been present for two years, without prior history of neoplastic or autoimmune disease. Physical examination revealed a mass located at the lower pole of the right parotid, with greatest diameter of about 4 cm, having a firm structure, not attached to adjacent structures, without right facial nerve involvement (Figure 7). Computed tomography with contrast material revealed a nodule of 33 mm greatest diameter located within the right parotid gland parenchyma, without direct contact with major vascular elements of the neck and without nearby enlarged lymph nodes. FNAC was suggestive of lymphoproliferative lesion, composed of numerous monomorphic lymphoid cells within a background of non-specific inflammatory cells (Figure 8).

**Figure 9:**
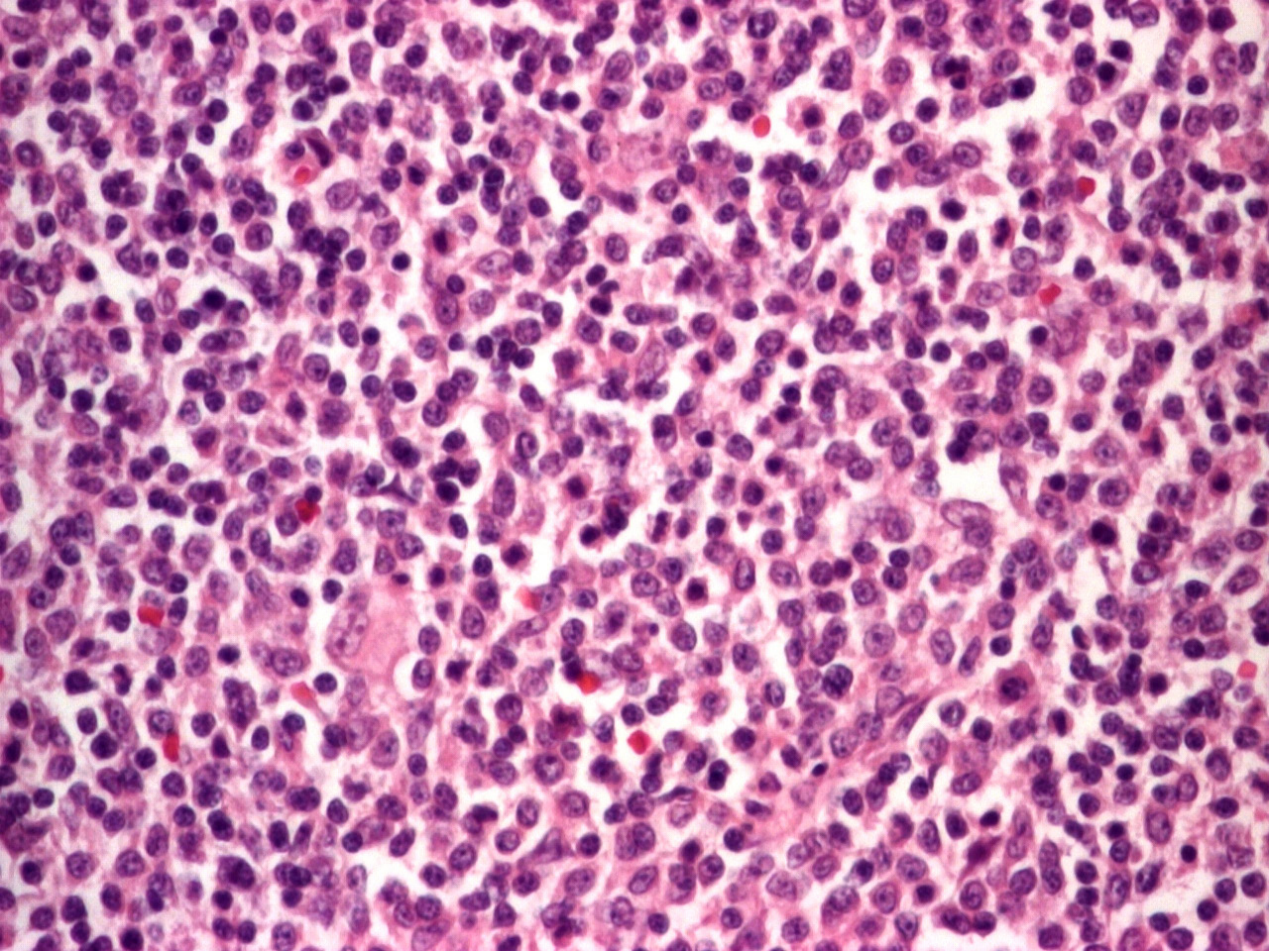
Marginal zone B-cell lymphoma, histology. No characteristic architectural feature of normal lymphoid tissue can be observed, monocytoid, large centrocyte-like B cells are observed. Tumor cells have moderately abundant pale cytoplasm, mostly irregular nuclei with clumped chromatin (ob. 10×, hematoxylin and eosin stain)

Superficial parotidectomy was performed with facial nerve preservation. Histology revealed a lymphoproliferative process consisting of small monomorphic cells, mostly displaying monocytoid and occasionally plasmacytoid features, present predominantly among follicles and within sinuses (Figure 9). Monocytoid lymphoid cells were CD20 positive B cells, within a background of CD3, CD5 positive reactive T lymphocytes. CD23 and CD10 expression highlighted residual germinal centers. Tumor cells showed low proliferative index (10%, as assessed by Ki67). Bcl-6, CyclinD, pancytokeratin were all negative. Based on histology and immunophenotype, a diagnosis of marginal zone lymphoma was made. The patient was referred to the Hematology Department, where he received specific oncologic treatment, including chemotherapy.

## Discussion

Tumors of the salivary gland in general, and malignant tumors in particular, frequently present as painless swelling associated with symptoms related to facial nerve palsy (in 10-15% of cases), pain (in 10-29% of patients), and fixation to deep structures. These clinical features are usually suggestive of local or regional tumor invasion^9^.

FNAC is an important method in the preoperative assessment of salivary gland lesions. Compared with excisional biopsy, it is easier to perform, safer, and has practically no complications^24^. Virtually, all head and neck masses, including salivary gland tumors may be assessed by FNAC, offering preoperative morphological assessment. Its less invasive nature makes it suitable for the pediatric population as well. Clinical studies have shown that aspiration cytology of salivary gland tumors has a sensitivity between 60-100% and a specificity between 90-100% in the diagnosis of these lesions^4^. Nevertheless, rare salivary gland tumors, including rare histological variants of common tumors, limit the diagnostic accuracy of FNAC^3^.

In rare malignant tumors that represent diagnostic challenges, FNAC alone may not be enough for adequate preoperative assessment. Moreover, sampling difficulties or other technical limitations may also hamper cytological diagnosis, including numerous histological variants and overlaps, making histological diagnosis often difficult. The use of immunohistochemistry greatly helps in diagnosis on such cases^26^. This study aimed to present some rare entities that may pose a diagnostic challenge during cytological evaluation.

Myoepithelial carcinoma is such a rare salivary gland tumor, consisting almost exclusively of tumor cells showing myoepithelial differentiation. It is clinically characterized by infiltrative growth and increased metastatic potential. Considered the malignant counterpart of myoepithelioma, most are in the parotid gland^5^. More than two thirds of the tumors with myoepithelial differentiation are benign^8^. It may reoccur, or on a pre-existing pleomorphic adenoma or myoepithelioma. Only a few clinical case series have been published so far^18^^,^^27^. Currently accepted diagnostic criteria include exclusive myoepithelial differentiation and tumor infiltration of surrounding soft tissues and salivary gland parenchyma^29^. Cytological features are diverse and not always include obvious signs of malignancy. There may be a plethora of cytological features, such as epithelioid, spindle, plasmacytoid, and even clear cells that were all described in FNAC smear obtained from such cases^10^^,^^29^.

In our case, despite the absence of specific features, unequivocal atypical nuclear features were present. Cytological diagnosis is difficult partly because of the presence of other lesions that may contain cells with myoepithelial differentiation^1^. Histology is definitive for the final diagnosis and, in combination with immunohistochemistry, aids in determining the myoepithelial differentiation of tumor cells. Most studies suggest alpha-smooth-muscle actin (SMA) and calponin as the most useful markers of myoepithelial differentiation. A combined use of these markers with others is recommended^14^. In our case, tumor cells expressed cytokeratin 5/6 and cytokeratin 7, SMA, S-100 protein, and p63, but they were negative for desmin.

The prognosis is variable with high rates of relapse and a propensity for distant metastasis. Thus, myoepithelial carcinoma is considered a high grade malignancy, with unpredictable behavior. Most tumors have an ability for extensive local infiltrative growth and destruction of nearby tissues^11^.

Oncocytic carcinoma is another rare neoplasm with less than 50 reported cases. It is considered a highly malignant neoplasm with tendency for local infiltrative growth and perineural invasion, frequent local metastasis in cervical lymph nodes and distant metastasis to central nervous system, bones, liver, and lungs^19^. It may pose a real challenge to the cytopathologist since oncocytic cellular change is not infrequent in salivary gland lesions, including reactive non-neoplastic changes. Such tumors include basal cell adenoma, pleomorphic adenoma, myoepithelioma, cystadenoma, canalicular adenoma, Warthin's tumor, acinar cell carcinoma, and mucoepidermoid carcinoma. However, a purely oncocytic tumor with associated nuclear features suggestive of malignancy should raise the possibility of oncocytic carcinoma^7^.

Cytological features were unspecific in our case, except that it highlighted oncocytic cells and nuclear features suggestive of malignancy.

Histological examination combined with immunohistochemistry offers a definitive diagnosis. Tumor cell are not only strongly reactive with mitochondrial antigen, but also express cytokeratin AE-1/AE-3, cytokeratin 7, CEA, EMA, and p63. The prognosis is difficult to assess due to the small number of published cases. Nevertheless, it is considered poor owing to frequent recurrences and early metastasis^5^.

Lymphoepithelial carcinoma represents less than 0.5% of malignant tumors of the salivary glands and is currently considered a variant of anaplastic carcinoma with characteristic dense lymphoid stroma^28^. Clinical studies have demonstrated a higher prevalence among the Southeast Asia and Arctic region native populations. Its link to Epstein-Barr virus infection was documented; however, this association does not show endemic characteristics in all cases^5^^,^^21^. Secondary determination of an undiagnosed mucosal lymphoepithelial carcinoma (usually nasal) should also be ruled out^2^.

Cytological features of our case were inconclusive for a certain diagnostic group, and it was labeled as a poorly differentiated malignant neoplasm with prominent reactive lymphoplasmocytic background. Histological diagnosis of lymphoepithelial carcinoma is partly based on the recognition of lymphoepithelial nests, similar to those present in benign lymphoepithelial lesion, displaying clear-cut atypia. Differential diagnosis is difficult due to overlapping features; it includes amelanotic melanoma, large cell lymphocytic and histiocytic neoplasms, and metastatic tumors affecting intraparotid lymph nodes as well. Undifferentiated lymphoepithelial carcinoma cells are cytokeratin positive, while lymphoid malignancies are negative, for cytokeratins and express leukocyte common antigen^22^. Lymphoepithelial carcinoma has a strong tendency to metastasize, first to the parotid nodes, followed by the upper cervical and retro-auricular lymph nodes, later to the supraclavicular and paratracheal nodes. Distant metastases usually involve the lungs, liver, bones, and brain. It has a better prognosis than other undifferentiated carcinomas of the salivary glands, perhaps because of the lymphoid stroma, which has a role in limiting the tumor's aggressiveness^28^.

Marginal zone lymphoma is an extra nodal low grade B-cell non-Hodgkin lymphoma that may affect the parotid gland. Other than the stomach, the salivary gland is one of the most common sites involved by extra nodal marginal zone lymphomas of mucosa-associated lymphoid tissue (MALT)^16^. Characteristically, it presents as a painless, progressively growing mass^13^. Most marginal zone lymphomas appear within the background of a chronic lymphoid infiltrate, possibly due to a long lasting inflammatory stimulus (such as benign lymphoepithelial lesion or Sjögren's syndrome), although it may present without evidence of previous condition^23^.

Primary lymphomas of the salivary gland are rare, about 4.7% of all lymphomas. Most are non-Hodgkin (MALT) lymphomas and occur in the parotid. Clinical presentation is nonspecific, therefore, preoperative assessment by FNAC is necessary. Lymphomas, in general, are difficult to diagnose by FNAC alone due to overlapping morphological features, lack of tissue architecture^13^. Nevertheless, the cytologist must be familiar with the cytological features of lymphomas, since therapy is different compared to an epithelial tumor^23^.

In our case, cytology suggested malignant lymphoproliferative lesion. Definitive diagnosis was obtained by histology, completed with immunohistochemistry. Marginal zone lymphoma diagnosis is difficult even on histology, and is based primary on excluding other lymphomas with specific markers. Tumor cells are positive for CD20 and CD79a or abnormal expression of CD43, and negative for CD5, CD10 and Cyclin D1. The prognosis of marginal zone lymphoma of the parotid gland is usually better than other extra nodal lymphomas. Even patients with advanced disease have relatively good survival^13^^,^^31^. Some studies found that prognosis is better in cases that arise in the background of Sjögren's syndrome^17^.

FNAC is a valuable diagnostic tool that offers the surgeon the ability to risk-stratify patients, to counsel them appropriately, and to avoid surgery in selected cases. Moreover, should FNAC confidently prove the existence of a benign process, facial nerve preservation must be attempted^20^. It is important to mention that, to maintain its high diagnostic accuracy as emphasized by Díaz, et al.^12^ (2014), all personnel involved in the process of salivary gland FNAC need to be continuously trained in this rather challenging area of diagnostic cytology^30^^,^^33^. Salivary gland cytology is an exciting branch of cytology that over the years has matured for the development of its own unified system for reporting, the Milan classification^25^.

## Conclusion

The primary role of FNAC in salivary gland tumors is to offer a morphological diagnosis whenever that is possible. This is possible in most cases; however, in rare neoplasms the cytological diagnosis is difficult, often being only indicative of the nature of the lesion rather than offering specific diagnosis. For cases with discrepancy between cytological and clinical impressions (especially in cases of rare malignant tumors, such as those presented herein), histological examination remains the gold standard.

Due to frequently overlapping histological features of various salivary gland neoplasms, the definitive diagnosis of rare salivary gland tumors (epithelial or non-epithelial) should always be made by histolopathology. Preoperative cytology is useful for the diagnostic triage of salivary masses but the precise diagnosis may sometimes be difficult to interpret by cytology alone.
